# Ten-year population trends of immunoglobulin use, burden of adult antibody deficiency and feasibility of subcutaneous immunoglobulin (SCIg) replacement in Hong Kong Chinese

**DOI:** 10.3389/fimmu.2022.984110

**Published:** 2022-12-14

**Authors:** Andy Ka Chun Kan, Garret Man Kit Leung, Valerie Chiang, Elaine Yuen Ling Au, Chak Sing Lau, Philip Hei Li

**Affiliations:** ^1^ Division of Rheumatology and Clinical Immunology, Department of Medicine, Queen Mary Hospital, The University of Hong Kong, Hong Kong, Hong Kong SAR, China; ^2^ Division of Haematology, Medical Oncology and Haemopoietic Stem Cell Transplantation, Department of Medicine, Queen Mary Hospital, The University of Hong Kong, Hong Kong, Hong Kong SAR, China; ^3^ Division of Clinical Immunology, Department of Pathology, Queen Mary Hospital, Hong Kong, Hong Kong SAR, China

**Keywords:** antibody deficiency, chinese, primary immunodeficiency disease, immunoglobulin therapy, subcutaneous, adult

## Abstract

**Background:**

Adult antibody deficiency remains under-recognised and under-studied – especially among Asian populations. Patterns of immunoglobulin use and the feasibility of subcutaneous immunoglobulin (SCIg) replacement among Chinese patients remains unclear.

**Objective:**

To investigate the trends of immunoglobulin use, burden of adult antibody deficiency and the outcomes of patients on SCIg compared to intravenous immunoglobulin (IVIg) replacement in Hong Kong through a retrospective observational study.

**Methods:**

Population-wide data of immunoglobulin recipients in Hong Kong between 2012 and 2021, and longitudinal clinical data of adult immunodeficiency patients at Queen Mary Hospital were collected and analysed.

**Results:**

Total immunoglobulin consumption and recurrent immunoglobulin recipients increased continuously from 175,512g to 298,514g (ρ=0.99, p<0.001) and 886 to 1,508 (ρ=0.89, p=0.001) between 2012-21 in Hong Kong. Among 469 immunoglobulin recipients at Queen Mary Hospital in 2021, 344 (73.3%) were indicated for replacement. Compared to those on IVIg (n=14), patients on SCIg replacement (n=8) had fewer immunodeficiency-related hospitalisations (IRR=0.11) and shorter duration of hospitalisation stay (IRR=0.10) per year, as well as better quality of life (SF-36v2 Health Survey and Life Quality Index). Estimated annual healthcare cost of SCIg replacement per patient was lower than that of IVIg (HKD196,850 [USD25,096] vs HKD222,136 [USD28,319]).

**Conclusion:**

There was a significantly increasing burden of adult antibody deficiency and immunoglobulin consumption in Hong Kong. SCIg was feasible and more cost-effective when compared to IVIg, with SCIg patients experiencing better clinical outcomes and quality of life. Future prospective studies to confirm the long-term efficacy and superiority of SCIg are required.

## 1 Introduction

Immunodeficiency among adults remains an important but severely under-recognised entity, with antibody deficiency being the most common subtype (1, 2). Antibody deficiencies can be classified as either primary or secondary – with secondary being much more common than their primary counterparts among adult patients (3, 4). Moreover, the prevalence of antibody deficiency continues to increase due to the popularising use of novel immunosuppressants and B-cell depleting therapies (e.g. anti-CD20 monoclonal antibodies such as rituximab) (3, 4). Although primary antibody deficiencies among paediatric patients have been extensively studied, the burden of adult antibody deficiency has not been well-characterised – especially among Asians and Chinese populations (1, 2, 5–10).

Normal human immunoglobulin, a pooled human blood product consisting of mainly IgG, has been used as a form of replacement in patients with antibody deficiency or as immunomodulation for various immune-mediated diseases (such as chronic inflammatory demyelinating polyradiculoneuropathy, multifocal motor neuropathy etc.) (11, 12). For replacement, immunoglobulin is usually administered with a relatively lower dose at regular intervals; while in immunomodulation, a relatively high dose is usually administered as single or short-term course (13–15). As a plasma-derived product from blood donors, immunoglobulin remains a precious and valuable resource especially during the Coronavirus disease (COVID-19) era with substantial declines in blood donations (16–18). Various resource-saving measures, such as establishment of immunoglobulin governance committees, have been implemented in many countries to optimise immunoglobulin use and stewardship (19–21). However, such measures do not currently exist in Hong Kong and the trends of immunoglobulin use remains unknown.

Traditionally, immunoglobulin has been administered intravenously (IVIg) or, uncommonly, intramuscularly. Subcutaneous immunoglobulin (SCIg) is a newer route of immunoglobulin replacement which was only introduced to Hong Kong since recently. In contrast to the IVIg which requires recurrent venous access and is administered during monthly hospital/day centre admissions, SCIg can be self-administrated by patients (or their carers) once every 1 to 2 weeks in the comfort of their own homes (22). SCIg has been shown to achieve at least comparable clinical outcomes compared with IVIg replacement, but with fewer systemic side effects and better health-related quality of life (HRQoL) (23–30). However, these findings have mostly been reported from Western cohorts, and the effectiveness and feasibility of SCIg replacement in Asia and among Chinese are unknown.

In view of these shortcomings, we took advantage of the availability of population-wide data and conducted this study to investigate the ten-year trends of immunoglobulin use and burden of adult antibody deficiency in Hong Kong. We also studied the feasibility of SCIg replacement by comparing the clinical outcomes and HRQoL of patients on SCIg and IVIg replacement in the real-world setting among Hong Kong Chinese.

## 2 Materials and methods

### 2.1 Study participants

In this retrospective observational cohort study, anonymised data were systematically retrieved from the Hong Kong Hospital Authority Clinical Data Analysis and Reporting System to identify patients of Hong Kong who received normal immunoglobulin between 2012 and 2021. The Hospital Authority is the sole publicly funded health care system in Hong Kong that serves a population of more than 7 million patients through 43 hospitals, 49 specialist outpatient clinics, and 73 general outpatient clinics. These facilities are organised into 7 clusters (Hong Kong East, Hong Kong West, Kowloon Central, Kowloon East, Kowloon West, New Territories East, and New Territories West) on the basis of geographical locations and provide approximately 90% of inpatient care in Hong Kong (31).

For subgroup analysis, longitudinal clinical data from all adult patients who received normal immunoglobulin and immunodeficiency patients who have attended the Immunology Clinic of Queen Mary Hospital (belonging to the Hong Kong West Cluster) were retrieved. Queen Mary Hospital remains the only centre with a Specialist in Immunology & Allergy and is responsible for the clinical care of immunodeficiency patients in Hong Kong. Therefore, this cohort likely represents the entire burden of adult immunodeficiency patients of the territory during the study period. Paediatric immunodeficiency patients and patients receiving immunoglobulin for non-immunodeficiency disorders (such as dermatological, haematological, neurological, rheumatological, etc.) would be seen throughout other geographical clusters of Hong Kong. As a tertiary referral centre and the only centre with a Specialist in Immunology & Allergy, Queen Mary Hospital also manages patients from other geographical clusters throughout the territory. However, not all patients receive immunoglobulin replacement in Queen Mary Hospital and may be referred to their nearest regional hospital (according to geographical location) for IVIg to facilitate patient convenience.

The study protocol was approved by the institutional review board of the University of Hong Kong and Hospital Authority Hong Kong West Cluster.

### 2.2 Whole-population trends of immunoglobulin use in Hong Kong

The total number of patients who received normal human immunoglobulin, as well as the number of courses per patient, amount and cost of normal immunoglobulin used in each year were recorded. Recurrent recipients were defined as patients who received at least two courses of normal immunoglobulin within one year (i.e. patients recurrently prescribed immunoglobulin as replacement therapy), while single-time recipients were defined as patients who received only one course of normal immunoglobulin within one year (i.e. patients prescribed ‘on-demand’ immunoglobulin as immunomodulatory therapy).

### 2.3 Burden of adult antibody deficiency in Hong Kong

Patients’ diagnoses and medication history were recorded. For patients who had received normal human immunoglobulin, their treatment indications were reviewed and categorised as either ‘replacement’ or ‘immunomodulation’) by a Specialist in Immunology & Allergy (11, 12).

### 2.4 Comparison between SCIg and IVIg

There were no local guidelines or recommendations regarding the choice between IVIg vs. SCIg during the study period. All adult immunodeficiency patients requiring immunoglobulin replacement during the study period were freely offered either route of administration. The final choice on route of administration was a jointly agreed decision between patients and Specialist in Immunology & Allergy. For the comparison between SCIg and IVIg, demographics and clinical data including immunodeficiency diagnosis, age of onset of immunodeficiency, age of diagnosis of immunodeficiency, clinical manifestations, serum immunoglobulin levels and treatment details were obtained. Serum immunoglobulin levels measured at the time of diagnosis were taken as baseline. Trough serum immunoglobulin levels were measured right before the latest immunoglobulin infusion. Clinical outcomes within one year before the study end date, including number of infection episodes, number of immunodeficiency-related and infection hospitalisation episodes, total duration of immunodeficiency-related and infection hospitalisations, as well as adverse events were recorded. Immunodeficiency-related hospitalisation was defined as hospitalisation due to infection or immunoglobulin replacement. HRQoL was assessed using the SF-36v2 Health Survey (SF-36v2) and Life Quality Index (LQI) during patients’ latest clinic visit (32–34). Validated English and Traditional Chinese (Hong Kong) SF-36v2 questionnaires were used (35–38). For LQI, the validated English questionnaire was translated into Traditional Chinese using forward-backward translation (**Table S1**).

An exploratory comparison of the annual healthcare cost of regular SCIg and IVIg replacement per patient was performed. The healthcare cost was the combined annual costs of normal immunoglobulin and immunodeficiency-related hospitalisation, using the immunoglobulin cost per gram, mean dose per body weight per month, mean body weight, median annual duration of immunodeficiency-related hospitalisation and mean daily cost of hospitalisation. All costs were expressed in Hong Kong Dollars (HKD) and United States Dollars (USD). Cost of SCIg per gram at Queen Mary Hospital (Hong Kong) was HKD530 (USD68), while that of IVIg per gram was HKD316 (USD40) during the study period. Mean daily cost of hospitalisation in Hong Kong was HKD6,020 (USD767) (39). In Hong Kong, all SCIg infusion equipment and consumables are provided by the suppliers without additional charge, and thus considered to be included as part of the SCIg drug cost.

### 2.5 Statistical analysis

All data were analysed using IBM SPSS Statistics 27.0. For continuous variables, normality of data was checked using the Shapiro–Wilk test. Data were presented as number, number (percentage), mean ± standard deviation or median (25^th^ percentile to 75^th^ percentile), as appropriate. Spearman’s correlation was used in the analysis of whole-population trends of immunoglobulin use. In the comparison of SCIg and IVIg, the distribution of demographics was examined using the chi-square test, Fisher exact test (if >5% of the cells have expected count <5), student’s T-test or Mann-Whitney U test, as appropriate. Multiple imputation with 20 imputed data sets was performed for the missing data of baseline laboratory values using the bar procedure (40). For these variables, multiple imputation was used for the main analyses and complete-case analysis was used for the sensitivity analyses. In all statistical tests, two-sided p-values <0.05 were considered significant.

## 3 Results

### 3.1 Continuous increase in immunoglobulin consumption over the past decade in Hong Kong, with majority being recurrent recipients

**Figure 1:**
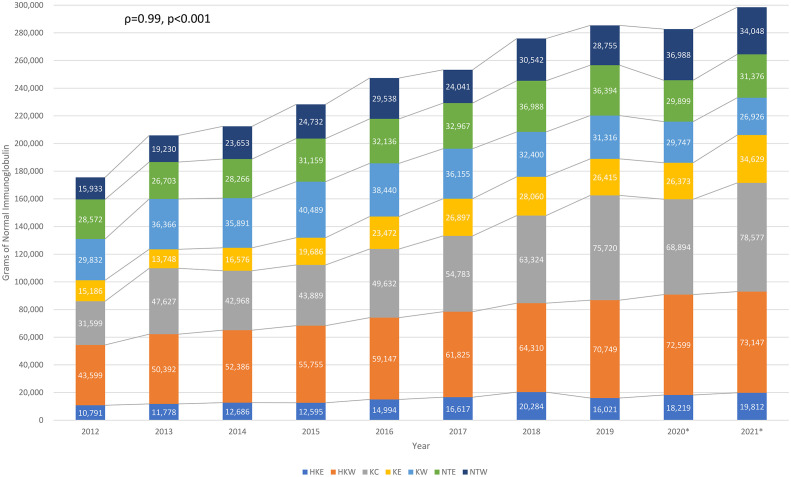
Longitudinal normal immunoglobulin consumption over the past decade in Hong Kong. HKE, Hong Kong East Cluster; HKW, Hong Kong West Cluster; KC, Kowloon Central Cluster; KE, Kowloon East Cluster; KW, Kowloon West Cluster; NTE, New Territories East Cluster; NTW, New Territories West Cluster. *Outbreak of COVID-19 in Hong Kong since January 2020, some of the healthcare services had been temporarily suspended upon waves of outbreak.

Total amount of immunoglobulin consumption in Hong Kong from 2012 to 2021, sorted by Hospital Authority clusters, are shown in **Figure 1**. The total amount of immunoglobulin consumption significantly increased from 175,512g to 298,514g (Spearman’s correlation coefficient [ρ]=0.99, p<0.001). The average immunoglobulin consumption increased from 128g to 159g per patient (ρ=0.86, p=0.003). The total number of immunoglobulin recipients and expenditure also increased continuously from 1,367 to 1,873 (ρ=0.71, p=0.022) (**Figure S1**) and from HKD55,541,992 (USD7,080,829) to HKD97,523,880 (USD12,432,939) from 2012 to 2021 (ρ=0.99, p<0.001) (**Figure S2**), respectively.

**Figure 2:**
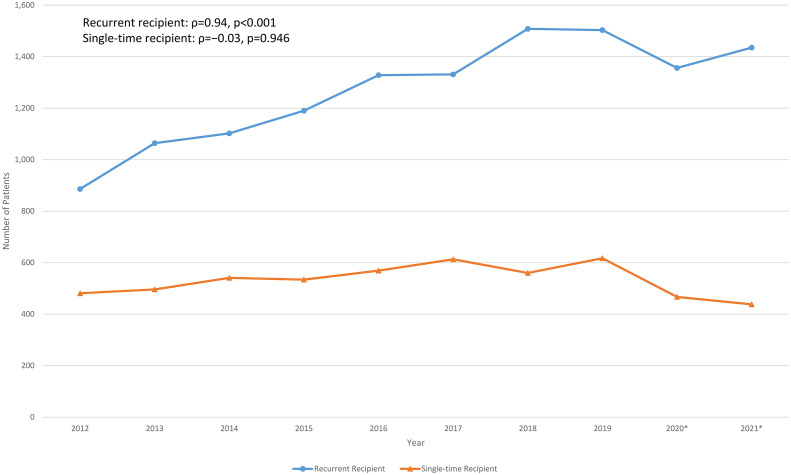
Longitudinal recurrent and single-time normal immunoglobulin recipients over the past decade in Hong Kong. *Outbreak of COVID-19 in Hong Kong since January 2020, some of the healthcare services had been temporarily suspended upon waves of outbreak.

The number of recurrent and single-time immunoglobulin recipients per year from 2012 to 2021 are shown in **Figure 2**. The number of recurrent recipients increased from 886 to 1,508 (ρ=0.89, p=0.001), while the number of single-time immunoglobulin recipients remained relatively stable (ρ=−0.03, p=0.946). The proportion of recurrent immunoglobulin recipients also increased from 65% in 2012 to 77% in 2021 (ρ=0.94, p<0.001).

### 3.2 Majority of immunoglobulin recipients were indicated for replacement, and most commonly for haematology patients

In subgroup analysis, 469 patients received immunoglobulin at Queen Mary Hospital (Hong Kong) in 2021; 344 (73.3%) received immunoglobulin for replacement, while the remaining 125 (26.7%) for immunomodulation. Among those receiving immunoglobulin replacement therapy, the most common were haematology patients (285, 82.8%), followed by solid organ transplant recipients (21, 6.1%) and primary immunodeficiencies (15, 4.4%). Among haematology patients, patients with history of lymphoma and post-haematopoietic stem-cell transplantation accounted for the majority with 36.1% and 33.7%, respectively (**Figure S3**).

### 3.3 Significant burden of antibody deficiency among adult immunodeficiency patients

**Figure 3:**
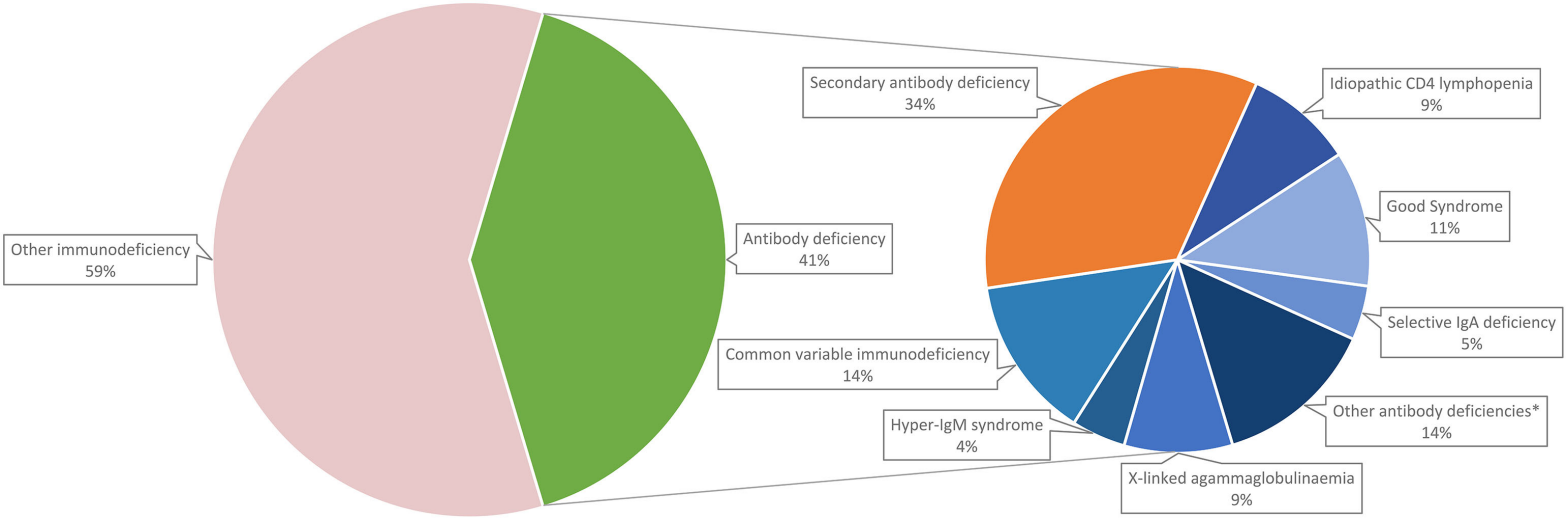
Breakdown of adult immunodeficiency patients in Hong Kong. *Other antibody deficiencies include specific antibody deficiency (2.3%), late-onset combined immunodeficiency (2.3%), ADA deficiency (2.3%), Wiskott-Aldrich syndrome (2.3%), chromosome 22q11.2 deletion syndrome (2.3%) and X-linked immunodeficiency with magnesium defect, Epstein-Barr virus (EBV) infection, and neoplasia (2.3%).

A total of 108 patients with confirmed immunodeficiencies attended the Immunology Clinic at Queen Mary Hospital (Hong Kong). Breakdown of their diagnoses is shown in **Figure 3**. Forty-four (40.7%) of the patients had antibody deficiency; among them, 29 (65.9%) had primary antibody deficiency and 15 (34.1%) had secondary antibody deficiency. Common variable immunodeficiency was the most common diagnosis among primary antibody deficiency (6, 20.7%).

### 3.4 Better clinical outcomes and HRQoL among SCIg compared to IVIg patients

A total of 22 antibody deficiency patients treated continuously with immunoglobulin replacement were included in the comparison of SCIg and IVIg replacement; 8 (36.4%) were on SCIg and 14 (63.6%) on IVIg. Their diagnoses are shown in **Table S2**. In this cohort, 16 (72.7%) and 6 (27.3%) of the patients had primary and secondary antibody deficiencies, respectively. **Table 1** summarises the clinical characteristics and treatment details of patients on SCIg and IVIg replacement. Demographics, aetiologies, manifestations, laboratory values, duration of immunoglobulin replacement and use of antimicrobial prophylaxis were similar between the two groups. Patients on SCIg required a significantly lower dose of immunoglobulin per body weight per month compared with patients on IVIg (0.52 ± 0.07 vs 0.66 ± 0.23 g/kg/month, p=0.046). Sensitivity analyses yielded similar results (**Table S3**).

**Table 1:** Demographics and clinical features of patients on regular IVIg and SCIg replacement.

Variables	All	IVIg	SCIg	p-value
N, %	22	14 (63.6)	8 (36.4)	
*Clinical characteristics*				
Male, n (%)	13 (59.1)	7 (50.0)	6 (75.0)	0.380
Age, years	48.2 ± 15.8	43.8 (38.8-58.8)	38.8 (29.9-71.9)	0.815
Age of onset, years	33.5 (4.50-57.8)	39.5 (4.50-53.8)	11.5 (4.00-66.0)	1.000
Age of diagnosis, years	38.9 ± 23.0	41.5 (27.4-56.8)	17.9 (14.5-67.4)	0.714
Aetiology, n (%)				0.624
Primary	16 (72.7)	11 (78.6)	5 (62.5)	
Secondary	6 (27.3)	3 (21.4)	3 (37.5)	
*Manifestations*				
Infection, n (%)	22 (100)	14 (100)	8 (100)	N/A
Autoimmune and/or autoinflammatory disease, n (%)	8 (36.4)	6 (42.9)	2 (25.0)	0.649
Malignancy, n (%)	9 (40.9)	6 (42.9)	3 (37.5)	1.000
*Laboratory values*				
Baseline IgG, mg/dL*	360 (67.5-653)	309 ± 264	490 ± 251	0.132
Baseline IgA, mg/dL*	36.4 (7.50-68.9)	14.5 (0.00-63.5)	50.0 (32.2-94.8)	0.165
Baseline IgM, mg/dL*	32.0 (4.50-240)	32.0 (0.00-337)	29.5 (7.50-224)	0.868
Trough IgG, mg/dL	1081 ± 202	1086 ± 236	1073 ± 135	0.890
Trough IgA, mg/dL	19.0 (0.00-68.3)	5.50 (0.00-59.0)	40.5 (0.00-99.5)	0.441
Trough IgM, mg/dL	0.00 (0.00-69.5)	19.5 (0.00-182)	0.00 (0.00-39.0)	0.188
*Treatment details*				
Duration of Ig replacement, years	3.10 (1.27-15.4)	1.59 (1.12-6.90)	15.4 (3.67-19.0)	0.059
Duration of current modality of Ig replacement, years	1.44 (1.02-1.83)	1.38 (1.00-3.60)	1.57 (1.04-1.72)	0.920
Stable dose of Ig replacement, g/kg/month	0.56 (0.46-0.79)	0.66 ± 0.23	0.52 ± 0.07	**0.046**
Antimicrobial prophylaxis, n (%)	15 (68.2)	11 (78.6)	4 (50.0)	0.343

Continuous data were presented as mean ± standard deviation or median (25th to 75th percentile). Categorical data were presented as number (percentage). IVIg, intravenous immunoglobulin; SCIg, subcutaneous immunoglobulin; Ig, immunoglobulin; N/A, not applicable. *Multiple imputation performed. **Bold** denotes statistical significance (p<0.05).

Comparisons of the infection and hospitalisation outcomes are shown in **Table 2**. Patients on SCIg had less frequent immunodeficiency-related hospitalisations compared to patients on IVIg (0.50 [0.00-1.75] vs 13.0 [12.0-14.3] episodes, incidence rate ratio [IRR]: 0.11). Patients on SCIg also had shorter annual duration of stay of immunodeficiency-related hospitalisation (0.50 [0.00-4.25] vs 13.0 [12.0-27.8] days, IRR: 0.10). The rate of reported adverse events were similar between the two groups (**Table S4**).

**Table 2:** Infection and hospitalisation outcomes of patients on regular IVIg and SCIg replacement.

Variables	Values	Incidence rate ratio (95% CI)
Infection in 1 year, episodes#		
Treatment modality		
IVIg	4.86 ± 4.37	Referent
SCIg	3.13 ± 3.09	0.64 (0.28-1.46)
Immunodeficiency-related hospitalisation in 1 year, episodes*		
Treatment modality		
IVIg	13.0 (12.0-14.3)	Referent
SCIg	0.50 (0.00-1.75)	0.11 (0.06-0.20)
Infection hospitalisation in 1 year, episodes#		
Treatment modality		
IVIg	0.00 (0.00-1.75)	Referent
SCIg	0.00 (0.00-0.75)	0.09 (0.02-1.29)
Total duration of stay of immunodeficiency-related hospitalisations in 1 year, days#		
Treatment modality		
IVIg	13.0 (12.0-27.8)	Referent
SCIg	0.50 (0.00-4.25)	0.10 (0.04-0.22)
Total duration of stay of infection hospitalisations in 1 year, days#		
Treatment modality		
IVIg	0.00 (0.00-9.75)	Referent
SCIg	0.00 (0.00-1.50)	0.10 (0.01-1.10)

Data were presented as mean ± standard deviation or median (25th to 75th percentile). IVIg, intravenous immunoglobulin; SCIg, subcutaneous immunoglobulin. *Poisson regression. #Negative binomial regression.

Comparison of the patients’ HRQoL is shown in **Table 3**. Patients on SCIg had better HRQoL in the Physical Component Summary of SF-36v2 compared with patients on IVIg replacement (54.5 ± 5.66 vs 45.8 ± 8.35). Patients on SCIg also had better immunoglobulin replacement-specific quality of life in the ‘Treatment Interference’ (88.9 ± 9.03 vs 64.3 ± 20.1) and ‘Therapy Setting’ (94.4 [90.3-98.6] vs 63.9 [54.2-94.4]) domains of LQI.

**Table 3:** Health-related quality of life of patients on regular IVIg and SCIg replacement.

Variables	All	IVIg	SCIg
N, %	22	14 (63.6)	8 (36.4)
*Quality of Life*			
SF-36v2 – Physical Functioning (PF)	95.0 (75.0-100)	90.0 (67.5-100)	100 (95.0-100)
SF-36v2 – Role-Physical (RP)	78.1 (56.3-100)	75.0 (56.3-89.1)	100 (62.5-100)
SF-36v2 – Bodily Pain (BP)	62.0 (51.0-100)	57.0 (48.5-66.5)	92.0 (57.5-100)
SF-36v2 – General Health (GH)	36.0 (25.0-63.0)	31.0 (25.0-53.3)	52.5 (25.8-80.8)
SF-36v2 – Vitality (VT)	56.3 ± 19.4	54.9 ± 16.1	58.6 ± 25.2
SF-36v2 – Social Functioning (SF)	75.0 (59.4-100)	68.8 (59.4-100)	87.5 (53.1-100)
SF-36v2 – Role-Emotional (RE)	75.0 (50.0-100)	75.0 (50.0-100)	87.5 (35.4-100)
SF-36v2 – Mental Health (MH)	71.8 ± 22.9	72.9 ± 19.1	70.0 ± 29.2
SF-36v2 – Physical Component Summary (PCS)	49.0 ± 8.52	45.8 ± 8.35	54.5 ± 5.66
SF-36v2 – Mental Component Summary (MCS)	46.4 ± 13.3	47.7 ± 11.2	44.3 ± 17.1
LQI – Treatment Interference	73.2 ± 20.6	64.3 ± 20.1	88.9 ± 9.03
LQI – Therapy-related Problems	75.0 (69.8-88.5)	75.0 (67.7-81.3)	85.4 (67.7-91.7)
LQI – Therapy Setting	83.3 (59.7-94.4)	63.9 (54.2-94.4)	94.4 (90.3-98.6)
LQI – Treatment Cost	95.8 (50.0-100)	87.5 (50.0-100)	100 (56.3-100)

Data were presented as mean ± standard deviation or median (25th to 75th percentile). IVIg, intravenous immunoglobulin; SCIg, subcutaneous immunoglobulin; SF-36v2, SF-36v2 Health Survey; LQI, Life Quality index.

### 3.5 Lower healthcare cost per patient for SCIg replacement

Using the mean weight of all patients on immunoglobulin replacement (62.2kg), the estimated annual healthcare costs of regular SCIg and IVIg replacement per patient were HKD196,850 (USD25,096) and HKD222,136 (USD28,319) respectively. Projecting from the costs saved from immunodeficiency-related hospitalisation costs alone, HKD25,286 (USD3,221) could be potentially saved per patient per year on SCIg compared to IVIg.

## 4 Discussion

To our knowledge, this is the first study to characterise the burden of adult antibody deficiency as well as comparing the outcomes of SCIg and IVIg replacement in the East and among Chinese patients. As the only dedicated unit responsible for managing adult immunodeficiency patients in Hong Kong, our cohort is highly representative of the entire immunodeficiency burden of the entire territory. In this retrospective observational study, we observed a substantial burden of adult antibody deficiency in Hong Kong Chinese, as well as better clinical outcomes and HRQoL among patients receiving SCIg replacement compared to those on IVIg.

It has been estimated that the global immunoglobulin consumption has been increasing at an average annual rate of 8% since 2010 (41). Studies in other populations reported increasing trends of immunoglobulin use before the era of COVID-19 (42–45). Similarly, our findings (including data during and following various COVID-19 outbreaks in Hong Kong in 2020) demonstrated significant increase in population-wide immunoglobulin consumption and expenditure over the past decade. This continuous rising trend persisted throughout the COVID-19 pandemic and was mainly among recurrent immunoglobulin recipients (for replacement), while the number of single-time immunoglobulin recipients (for immunomodulation) remained relatively stable (14, 15). Together with the overall increasing immunoglobulin usage and demand in Hong Kong, our findings suggest that there is a steadily growing burden of antibody deficiency in our population. Interestingly, trends in increase immunoglobulin consumption were consistent throughout the 7 geographical clusters of Hong Kong, but most marked in Kowloon Central (2.5x increase) and Kowloon East (2.3x increase). Reasons for disparate increases between geographical regions are likely multifactorial, including disproportionate changes in population densities and demographics, service expansion of immunological/haematological-based specialties or socio-economic variations between different clusters over the past decade. Dedicated studies regarding potential inequalities in prescription and their causes would be of great interest.

In subgroup analysis, we identified that around three quarters of adult immunoglobulin recipients were given immunoglobulin as replacement therapy, which was comparable to findings of our population-wide analysis. Among those indicated for immunoglobulin replacement, the overwhelming majority (96%) had secondary antibody deficiency (most patients being haematology patients), while only 4% had primary immunodeficiency. This supports the observation that there is a much higher prevalence of secondary antibody deficiencies compared to primary antibody deficiencies (4). Furthermore, the indications for immunoglobulin replacement varied across different studies and time. More recent studies tend to report that secondary antibody deficiency are more common than primary antibody deficiency (19, 45), whereas older studies reported the opposite (46, 47). We postulate that this reflects the progressive shift of the burden from primary to secondary antibody deficiencies in recent years.

When comparing between different routes of immunoglobulin replacement in this pragmatic study of real-worldpractice, we observed reduced immunodeficiency-related hospitalisations and duration of stay, as well as better HRQoL among adult patients on SCIg replacement than IVIg. The main difference in hospitalisation rates arose from fewer hospital stays for IVIg administration. These findings are similar to the experience reported by previous studies in other populations (23–30). In addition, we also identified that patients on SCIg required lower average dose of immunoglobulin compared to those on IVIg, while achieving similar infection outcomes. This is contrary to earlier recommendations in the United States, which suggested the use of higher doses (IVIg : SCIg = 1:1.37 or 1:1.53) when calculating the dose of SCIg replacement; while European recommendations suggest the use of equivalent doses (IVIg : SCIg = 1:1) (48–51). Further studies will be required to investigate whether his phenomenon is ethnic- or region-specific. Nonetheless, we propose that in Hong Kong Chinese, SCIg can be considered as a more effective route of immunoglobulin replacement and that we could potentially save on our precious plasma-derived resources by optimising more SCIg use.

Furthermore, we also demonstrated that SCIg is more cost-effective than IVIg as a form of immunoglobulin replacement for adult patients in Hong Kong. Our finding is similar to that of other cost-effective analysis studies in Australia, Canada, Iran, Spain, France, Japan and Switzerland (52–58). Projecting from the costs saved from immunodeficiency-related hospitalisation costs alone, we could potentially save HKD25,286 (USD3,221) per patient per year on SCIg compared to IVIg. This figure is likely an under-estimate, as we did not include indirect costs such as time-off work and productivity lost used for admissions for IVIg, need for healthcare staff during IVIg administration and difference in quality of life. Therefore, we believe that SCIg may be a superior alternative to IVIg replacement among adult patients in Hong Kong, in terms of clinical outcomes, HRQoL and overall healthcare costs. Unfortunately, as of the study period, SCIg remains unsubsidised in Hong Kong and inaccessible by most patients due to financial concerns. Furthermore, unlike in some countries, home administration of IVIg is not recommended in Hong Kong. We therefore strongly advocate for wider adoption and subsidisation of SCIg in Hong Kong.

This study has several limitations. First, immunoglobin use only indirectly reflected the burden of antibody deficiency and milder forms of antibody deficiency which did not warrant immunoglobulin replacement may have been missed. Hence, the actual burden of antibody deficiency may be even larger. Furthermore, there were also numerically a higher proportion of IVIg-treated patients with autoimmune/autoinflammatory disorders (which require higher dosing of immunoglobulin) and SCIg patients had longer duration of overall immunoglobulin replacement (which may have led to better tailoring of dosing regimen), although both variables did not reach statistical significance. Second, the small number of patients in the immunoglobulin replacement cohort did not allow for multivariable statistical analysis. Third, in this descriptive, pragmatic study we aimed to compare SCIg and IVIg replacement among adult antibody deficiency patients; in this pragmatic study, and the drug efficacy was not directly studied. It is possible that differences in real-world clinical practice also contributed to our results, rather than purely differences between the two administration routes alone. Fourth, clinical outcomes were assessed over one year and HRQoL was assessed at one time-point only. Long-term outcomes could not be studied, and HRQoL might vary over time. Fifth, the comparison of annual healthcare cost of SCIg and IVIg replacement per patient was only exploratory. Future formal cost-effectiveness analysis of SCIg replacement in Hong Kong is therefore warranted. Sixth, this was a retrospective observational study and firm conclusions on drug efficacy can not drawn. This highlights the urgent need for dedicated prospective studies in the future. Lastly, subgroup analysis and the comparative analysis between SCIg and IVIg were only performed on adult patients and do not pertain to paediatric immunodeficiency patients and their immunoglobulin use.

In conclusion, there has been significantly increasing immunoglobulin demand in Hong Kong over the past decade. The burden of adult antibody deficiency in Hong Kong is substantial but under-recognised, characterised by the dominance of secondary antibody deficiency with haematological diseases as the most common cause. Around 40% of adult immunodeficiency patients have antibody deficiency, but most patients, especially those with secondary antibody deficiency, did not receive immunologist care. In this retrospective observational study, adult antibody deficiency patients on SCIg replacement had better clinical outcomes and HRQoL compared to those on IVIg. Despite higher drug costs, we demonstrate that SCIg was overall more cost-effective than IVIg among adult patients with antibody deficiency. Although future studies will be required to draw conclusive evidence on the long-term efficacy of SCIg, we strongly advocate for wider adoption and subsidisation of SCIg in Hong Kong.

## Data availability statement

The original contributions presented in the study are included in the article/**Supplementary Material**. Further inquiries can be directed to the corresponding author.

## Ethics statement

The studies involving human participants were reviewed and approved by the institutional review board of the University of Hong Kong and Hospital Authority Hong Kong West Cluster. Written informed consent for participation was not required for this study in accordance with the national legislation and the institutional requirements.

## Author contributions

AK researched the data, performed statistical analyses and wrote the manuscript. GL, VC and EA reviewed the data and edited the manuscript. CL and PL supervised the project and critically reviewed and edited the manuscript. All authors contributed to the article and approved the submitted version.

## References

[1] Rosenberg E Dent PB Denburg JA. Primary immune deficiencies in the adult: A previously underrecognized common condition. J Allergy Clin Immunol Pract (2016) 4(6):1101–7. doi: 10.1016/j.jaip.2016.09.004 27836059

[2] Mansouri D Adimi P Mirsaedi M Mansouri N Tabarsi P Amiri M. Primary immune deficiencies presenting in adults: Seven years of experience from Iran. J Clin Immunol (2005) 25(4):385–91. doi: 10.1007/s10875-005-4124-0 16133995

[3] Li PH Lau CS. Secondary antibody deficiency and immunoglobulin replacement. Hong Kong Bull Rheum (2017) 17(1):1–5. doi: 10.1515/hkbrd-2017-0001

[4] Patel SY Carbone J Jolles S. The expanding field of secondary antibody deficiency: Causes, diagnosis, and management. Front Immunol (2019) 10:33. doi: 10.3389/fimmu.2019.00033 30800120 PMC6376447

[5] Takada H. Primary immunodeficiency in japan; epidemiology, diagnosis, and pathogenesis. Pediatr Int (2013) 55(6):671–4. doi: 10.1111/ped.12224 24112462

[6] Lim DL Thong BY Ho SY Shek LP Lou J Leong KP. Primary immunodeficiency diseases in Singapore – the last 11 years. Singapore Med J (2003) 44(11):579–86. 15007498

[7] Lee WI Huang JL Jaing TH Shyur SD Yang KD Chien YH. Distribution, clinical features and treatment in Taiwanese patients with symptomatic primary immunodeficiency diseases (Pids) in a nationwide population-based study during 1985-2010. Immunobiology (2011) 216(12):1286–94. doi: 10.1016/j.imbio.2011.06.002 21782277

[8] Sigstad HM Stray-Pedersen A Frøland SS. Coping, quality of life, and hope in adults with primary antibody deficiencies. Health Qual Life Outcomes (2005) 3:31. doi: 10.1186/1477-7525-3-31 15871746 PMC1177979

[9] Lam DS Lee TL Chan KW Ho HK Lau YL. Primary immunodeficiency in Hong Kong and the use of genetic analysis for diagnosis. Hong Kong Med J (2005) 11(2):90–6. 15815061

[10] Duraisingham SS Buckland M Dempster J Lorenzo L Grigoriadou S Longhurst HJ. Primary vs. secondary antibody deficiency: Clinical features and infection outcomes of immunoglobulin replacement. PloS One (2014) 9(6):e100324. doi: 10.1371/journal.pone.0100324 24971644 PMC4074074

[11] Nagelkerke SQ Kuijpers TW. Immunomodulation by ivig and the role of fc-gamma receptors: Classic mechanisms of action after all? Front Immunol (2014) 5:674. doi: 10.3389/fimmu.2014.00674 25653650 PMC4301001

[12] Sewell WA Jolles S. Immunomodulatory action of intravenous immunoglobulin. Immunology (2002) 107(4):387–93. doi: 10.1046/j.1365-2567.2002.01545.x PMC1782817 12460182

[13] Negi VS Elluru S Sibéril S Graff-Dubois S Mouthon L Kazatchkine MD. Intravenous immunoglobulin: An update on the clinical use and mechanisms of action. J Clin Immunol (2007) 27(3):233–45. doi: 10.1007/s10875-007-9088-9 17351760

[14] Jolles S Sewell WA Misbah SA. Clinical uses of intravenous immunoglobulin. Clin Exp Immunol (2005) 142(1):1–11. doi: 10.1111/j.1365-2249.2005.02834.x 16178850 PMC1809480

[15] Hartung HP Mouthon L Ahmed R Jordan S Laupland KB Jolles S. Clinical applications of intravenous immunoglobulins (Ivig)–beyond immunodeficiencies and neurology. Clin Exp Immunol (2009) 158 Suppl 1(Suppl 1):23–33. doi: 10.1111/j.1365-2249.2009.04024.x 19883421 PMC2801038

[16] Berger M. Principles of and advances in immunoglobulin replacement therapy for primary immunodeficiency. Immunol Allergy Clin North Am (2008) 28(2):413–37. doi: 10.1016/j.iac.2008.01.008 PMC7127239 18424340

[17] Chiem C Alghamdi K Nguyen T Han JH Huo H Jackson D. The impact of covid-19 on blood transfusion services: A systematic review and meta-analysis. Transfus Med Hemother (2021) 30(2):1–12. doi: 10.1159/000519245 34934412 PMC8678226

[18] Al-Riyami AZ Burnouf T Wood EM Devine DV Oreh A Apelseth TO. International society of blood transfusion survey of experiences of blood banks and transfusion services during the covid-19 pandemic. Vox Sang (2022) 117(6):822–30. doi: 10.1111/vox.13256 PMC9115426 35262978

[19] Rocchio MA Schurr JW Hussey AP Szumita PM. Intravenous immune globulin stewardship program at a tertiary academic medical center. Ann Pharmacother (2017) 51(2):135–9. doi: 10.1177/1060028016673071 27758967

[20] Tsapepas D Der-Nigoghossian C Patel K Berger K Vawdrey DK Salmasian H. Medication stewardship using computerized clinical decision support: A case study on intravenous immunoglobulins. Pharmacol Res Perspect (2019) 7(5):e00508. doi: 10.1002/prp2.508 31485333 PMC6715593

[21] Derman BA Schlei Z Parsad S Mullane K Knoebel RW. Changes in intravenous immunoglobulin usage for hypogammaglobulinemia after implementation of a stewardship program. JCO Oncol Pract (2021) 17(3):e445–e53. doi: 10.1200/op.20.00312 PMC8257910 32822257

[22] Skoda-Smith S Torgerson TR Ochs HD. Subcutaneous immunoglobulin replacement therapy in the treatment of patients with primary immunodeficiency disease. Ther Clin Risk Manag (2010) 6:1–10. doi: 10.1057/rm.2009.17 20169031 PMC2817783

[23] Lingman-Framme J Fasth A. Subcutaneous immunoglobulin for primary and secondary immunodeficiencies: An evidence-based review. Drugs (2013) 73(12):1307–19. doi: 10.1007/s40265-013-0094-3 23861187

[24] Wasserman RL Irani AM Tracy J Tsoukas C Stark D Levy R. Pharmacokinetics and safety of subcutaneous immune globulin (Human), 10% Caprylate/Chromatography purified in patients with primary immunodeficiency disease. Clin Exp Immunol (2010) 161(3):518–26. doi: 10.1111/j.1365-2249.2010.04195.x PMC2962970 20550549

[25] Desai SH Chouksey A Poll J Berger M. A pilot study of equal doses of 10% igiv given intravenously or subcutaneously. J Allergy Clin Immunol (2009) 124(4):854–6. doi: 10.1016/j.jaci.2009.07.051 19767071

[26] Wasserman RL Melamed I Kobrynski L Strausbaugh SD Stein MR Sharkhawy M. Efficacy, safety, and pharmacokinetics of a 10% liquid immune globulin preparation (Gammagard liquid, 10%) administered subcutaneously in subjects with primary immunodeficiency disease. J Clin Immunol (2011) 31(3):323–31. doi: 10.1007/s10875-011-9512-z 21424824

[27] Nicolay U Kiessling P Berger M Gupta S Yel L Roifman CM. Health-related quality of life and treatment satisfaction in north American patients with primary immunedeficiency diseases receiving subcutaneous igg self-infusions at home. J Clin Immunol (2006) 26(1):65–72. doi: 10.1007/s10875-006-8905-x 16418804

[28] Compagno N Cinetto F Semenzato G Agostini C. Subcutaneous immunoglobulin in lymphoproliferative disorders and rituximab-related secondary hypogammaglobulinemia: A single-center experience in 61 patients. Haematologica (2014) 99(6):1101–6. doi: 10.3324/haematol.2013.101261 PMC4040915 24682509

[29] Windegger TM English J Weston H Morwood K Kynn M Scuffham P. Longitudinal study of intravenous versus subcutaneous immunoglobulin replacement therapy in hematological malignancy. Asia Pac J Clin Oncol (2021) 17(6):546–54. doi: 10.1111/ajco.13515 33460509

[30] Windegger TM Lambooy CA Hollis L Morwood K Weston H Fung YL. Subcutaneous immunoglobulin therapy for hypogammaglobulinemia secondary to malignancy or related drug therapy. Transfus Med Rev (2017) 31(1):45–50. doi: 10.1016/j.tmrv.2016.06.006 27450021

[31] Hospital Authority. Hospital authority annual report (2018). Available at: https://www.ha.org.hk/haho/ho/cc/HA_Annual_Report_2020-21_en.pdf.

[32] Maruish ME. User’s manual for the sf-36v2 health survey. Lincoln, RI: QualityMetric Incorporated (2011).

[33] Daly PB Evans JH Kobayashi RH Kobayashi AL Ochs HD Fischer SH. Home-based immunoglobulin infusion therapy: Quality of life and patient health perceptions. Ann Allergy (1991) 67(5):504–10. 1958004

[34] Nicolay U Haag S Eichmann F Herget S Spruck D Gardulf A. Measuring treatment satisfaction in patients with primary immunodeficiency diseases receiving lifelong immunoglobulin replacement therapy. Qual Life Res (2005) 14(7):1683–91. doi: 10.1007/s11136-005-1746-x 16119180

[35] Brazier JE Harper R Jones NM O'Cathain A Thomas KJ Usherwood T. Validating the sf-36 health survey questionnaire: New outcome measure for primary care. BMJ (1992) (6846) 305:160–4. doi: 10.1136/bmj.305.6846.160 PMC1883187 1285753

[36] Jenkinson C Stewart-Brown S Petersen S Paice C. Assessment of the sf-36 version 2 in the united kingdom. J Epidemiol Community Health (1999) 53(1):46–50. doi: 10.1136/jech.53.1.46 10326053 PMC1756775

[37] Lam CL Gandek B Ren XS Chan MS. Tests of scaling assumptions and construct validity of the Chinese (Hk) version of the sf-36 health survey. J Clin Epidemiol (1998) 51(11):1139–47. doi: 10.1016/s0895-4356(98)00105-x 9817131

[38] Lam ETP Lam CLK Lo YYC Gandek B. Psychometrics and population norm of the Chinese (Hk) sf-36 health Survey_Version 2. HK Prac (2008) 30:185–98.

[39] Food and Health Bureau of the Government of the Hong Kong Special Administrative Region. Average cost (General (Acute & convalescent)) per patient day for each major specialty by hospital cluster for 2019-20 (2021). Available at: https://www.fhb.gov.hk/download/legco/replies/210413_sfc/fhb-h-e.pdf.

[40] Baranzini D. The “Bar procedure”: Spss single dataframe aggregating spss multiply imputed split files. (2018). doi: 10.13140/RG.2.2.33750.70722.

[41] Prevot J Jolles S. Global immunoglobulin supply: Steaming towards the iceberg? Curr Opin Allergy Clin Immunol (2020) 20(6):557–64. doi: 10.1097/aci.0000000000000696 PMC7752222 33044340

[42] Murphy MSQ Tinmouth A Goldman M Chassé M Colas JA Saidenberg E. Trends in ivig use at a tertiary care Canadian center and impact of provincial use mitigation strategies: 10-year retrospective study with interrupted time series analysis. Transfusion (2019) 59(6):1988–96. doi: 10.1111/trf.15271 30916409

[43] Hsu LI Chen JW Lin DT Hung YS Hou SM. Clinical use of intravenous immunoglobulin in Taiwan: A 10-year population study. J Formos Med Assoc (2021) 120(10):1921–5. doi: 10.1016/j.jfma.2021.02.017 33726936

[44] Modell V Quinn J Orange J Notarangelo LD Modell F. Primary immunodeficiencies worldwide: An updated overview from the Jeffrey modell centers global network. Immunol Res (2016) 64(3):736–53. doi: 10.1007/s12026-016-8784-z 26802037

[45] Shemer A Kivity S Shoenfeld Y. Clinical indications for intravenous immunoglobulin utilization in a tertiary medical center: A 9-year retrospective study. Transfusion (2018) 58(2):430–8. doi: 10.1111/trf.14427 29193136

[46] Ruiz-Antorán B Agustí Escasany A Vallano Ferraz A Danés Carreras I Riba N Mateu Escudero S. Use of non-specific intravenous human immunoglobulins in Spanish hospitals; need for a hospital protocol. Eur J Clin Pharmacol (2010) 66(6):633–41. doi: 10.1007/s00228-010-0800-y 20204337

[47] Constantine MM Thomas W Whitman L Kahwash E Dolan S Smith S. Intravenous immunoglobulin utilization in the Canadian Atlantic provinces: A report of the Atlantic collaborative intravenous immune globulin utilization working group. Transfusion (2007) 47(11):2072–80. doi: 10.1111/j.1537-2995.2007.01400.x 17958537

[48] Wasserman RL Melamed I Nelson RP Jr. Knutsen AP Fasano MB Stein MR. Pharmacokinetics of subcutaneous Igpro20 in patients with primary immunodeficiency. Clin Pharmacokinet (2011) 50(6):405–14. doi: 10.2165/11587030-000000000-00000 21553933

[49] Haddad E Berger M Wang EC Jones CA Bexon M Baggish JS. Higher doses of subcutaneous igg reduce resource utilization in patients with primary immunodeficiency. J Clin Immunol (2012) 32(2):281–9. doi: 10.1007/s10875-011-9631-6 PMC3305876 22193916

[50] Krishnarajah G Lehmann JK Ellman B Bhak RH DerSarkissian M Leader D Jr.. Evaluating dose ratio of subcutaneous to intravenous immunoglobulin therapy among patients with primary immunodeficiency disease switching to 20% subcutaneous immunoglobulin therapy. Am J Manag Care (2016) 22(15 Suppl):s475–s81. 27849353

[51] Jolles S Bernatowska E de Gracia J Borte M Cristea V Peter HH. Efficacy and safety of hizentra(®) in patients with primary immunodeficiency after a dose-equivalent switch from intravenous or subcutaneous replacement therapy. Clin Immunol (2011) 141(1):90–102. doi: 10.1016/j.clim.2011.06.002 21705277

[52] Windegger TM Nghiem S Nguyen KH Fung YL Scuffham PA. Cost-utility analysis comparing hospital-based intravenous immunoglobulin with home-based subcutaneous immunoglobulin in patients with secondary immunodeficiency. Vox Sang (2019) 114(3):237–46. doi: 10.1111/vox.12760 30883804

[53] Windegger TM Nghiem S Nguyen KH Fung YL Scuffham PA. Primary immunodeficiency disease: A cost-utility analysis comparing intravenous vs subcutaneous immunoglobulin replacement therapy in Australia. Blood Transfus (2020) 18(2):96–105. doi: 10.2450/2029.0083-19 32271703 PMC7141942

[54] Shabaninejad H Asgharzadeh A Rezapour A Rezaei N. Cost-effectiveness analysis of subcutaneous immunoglobulin replacement therapy in Iranian patients with primary immunodeficiencies. Med J Islam Repub Iran (2017) 31:94. doi: 10.14196/mjiri.31.94 29951395 PMC6014784

[55] Alsina L Montoro JB Moral PM Neth O Pica MO Sánchez-Ramón S. Cost-minimization analysis of immunoglobulin treatment of primary immunodeficiency diseases in Spain. Eur J Health Econ (2022) 23(3):551–8. doi: 10.1007/s10198-021-01378-x PMC8964571 34546485

[56] Beauté J Levy P Millet V Debré M Dudoit Y Le Mignot L. Economic evaluation of immunoglobulin replacement in patients with primary antibody deficiencies. Clin Exp Immunol (2010) 160(2):240–5. doi: 10.1111/j.1365-2249.2009.04079.x PMC2857947 20041884

[57] Igarashi A Kanegane H Kobayashi M Miyawaki T Tsutani K. Cost-minimization analysis of Igpro20, a subcutaneous immunoglobulin, in Japanese patients with primary immunodeficiency. Clin Ther (2014) 36(11):1616–24. doi: 10.1016/j.clinthera.2014.08.007 25236916

[58] Perraudin C Bourdin A Spertini F Berger J Bugnon O. Switching patients to home-based subcutaneous immunoglobulin: An economic evaluation of an interprofessional drug therapy management program. J Clin Immunol (2016) 36(5):502–10. doi: 10.1007/s10875-016-0288-z 27139500

