# Supplementary material for: Ten-year population trends of immunoglobulin use, burden of adult antibody deficiency and feasibility of subcutaneous immunoglobulin (SCIg) replacement in Hong Kong Chinese
Source: Front Immunol. 2022 Dec 14;13:984110. doi: 10.3389/fimmu.2022.984110 (PMC9795180; doi:10.3389/fimmu.2022.984110)

Supplementary Material

# Supplementary Tables

Table S1 Life Quality Index questionnaire (Traditional Chinese)

| 我的免疫球蛋白治療： | | | | | | | | | | | |
| --- | --- | --- | --- | --- | --- | --- | --- | --- | --- | --- | --- |
| 1. | 方便 | = | 7 | 6 | 5 | 4 | 3 | 2 | 1 | = | 不方便 |
| 2. | 不令我感到疼痛 | = | 7 | 6 | 5 | 4 | 3 | 2 | 1 | = | 令我感到疼痛 |
| 3. | 改善了我的健康 | = | 7 | 6 | 5 | 4 | 3 | 2 | 1 | = | 沒有改善我的健康 |
| 4. | 沒有阻礙我的 社交/家庭生活 | = | 7 | 6 | 5 | 4 | 3 | 2 | 1 | = | 阻礙我的 社交/家庭生活 |
| 5. | 沒有阻礙我 工作/上學 | = | 7 | 6 | 5 | 4 | 3 | 2 | 1 | = | 阻礙我工作/上學 |
| 6. | 在一個我感到舒服的地方進行 | = | 7 | 6 | 5 | 4 | 3 | 2 | 1 | = | 在一個我感到不舒服的地方進行 |
| 7. | 進行前所需的等候時間不太長 | = | 7 | 6 | 5 | 4 | 3 | 2 | 1 | = | 進行前所需的等候時間太長 |
| 8. | 在一個令人愉快的氣氛下進行 | = | 7 | 6 | 5 | 4 | 3 | 2 | 1 | = | 在一個令人不快的氣氛下進行 |
| 9. | 我認為是值得的 | = | 7 | 6 | 5 | 4 | 3 | 2 | 1 | = | 我認為是浪費時間的 |
| 10. | 不會令我感到焦慮或緊張 | = | 7 | 6 | 5 | 4 | 3 | 2 | 1 | = | 令我感到焦慮或緊張 |
| 11. | 就我而言不太昂貴 | = | 7 | 6 | 5 | 4 | 3 | 2 | 1 | = | 就我而言太過昂貴 |
| 12. | 不會令我過度依賴別人 | = | 7 | 6 | 5 | 4 | 3 | 2 | 1 | = | 會令我過度依賴別人 |
| 13. | 只需很少交通時間和成本 | = | 7 | 6 | 5 | 4 | 3 | 2 | 1 | = | 需要很多交通時間和成本 |
| 14. | 沒有限制我旅行或搬遷 | = | 7 | 6 | 5 | 4 | 3 | 2 | 1 | = | 限制我旅行或搬遷 |
| 15. | 進行的日期和時間是按我的方便來安排的 | = | 7 | 6 | 5 | 4 | 3 | 2 | 1 | = | 進行的日期和時間並非按我的方便來安排的 |

Table S2 Diagnosis of patients on regular IVIg and SCIg replacement

| **Diagnosis** | **IVIg** | **SCIg** |
| --- | --- | --- |
| X-linked agammaglobulinaemia | 1 (7.1%) | 3 (37.5%) |
| Common variable immunodeficiency | 3 (21.4%) | 2 (25.0%) |
| Hyper-IgM syndrome | 2 (14.3%) | 0 (0.0%) |
| ADA deficiency | 1 (7.1%) | 0 (0.0%) |
| Good Syndrome | 3 (21.4%) | 0 (0.0%) |
| X-linked immunodeficiency with Mg^2+^ defect (MAGT1 deficiency) | 1 (7.1%) | 0 (0.0%) |
| Secondary antibody deficiency* | 3 (21.4%) | 3 (37.5%) |
| Total | 14 | 8 |

*2 patients had autoimmune disease with immunosuppressant use, 2 patients had haematological malignancy (post-chemotherapy), 2 patients had solid organ tumour (post-chemotherapy)

Data were presented as number (percentage).

IVIg, intravenous immunoglobulin; SCIg, subcutaneous immunoglobulin; ADA, adenosine deaminase; MAGT1, Magnesium Transporter 1.

Table S3 Sensitivity analyses of variables with missing values in the comparison of SCIg and IVIg replacement (complete-case analyses)

| **Variables** | **All** | **IVIg** | **SCIg** | **p-value** |
| --- | --- | --- | --- | --- |
| N, % | 17 | 12 (70.6) | 5 (29.4) |  |
| *Laboratory values* |  |  |  |  |
| Baseline IgG, mg/dL | 321 (0.00-515) | 239±209 | 485±315 | 0.075 |
| Baseline IgA, mg/dL | 34.0 (0.00-83.0) | 14.5 (0.00-66.5) | 55.0 (22.5-136) | 0.195 |
| Baseline IgM, mg/dL | 18.0 (0.00-48.5) | 26.5 (0.00-143) | 12.0 (3.00-29.5) | 0.442 |

Data were presented as mean±standard deviation or median (25^th^ to 75^th^ percentile).

IVIg, intravenous immunoglobulin; SCIg, subcutaneous immunoglobulin; Ig, immunoglobulin.

Table S4 Adverse events of patients on regular IVIg and SCIg replacement

| **Variable** | **All** | **IVIg** | **SCIg** | **p-value** |
| --- | --- | --- | --- | --- |
| N, % | 22 | 14 (63.6) | 8 (36.4) |  |
| *Adverse events* |  |  |  |  |
| Adverse event in 1 year, n (%) | 15 (68.2) | 9 (64.3) | 6 (75.0) | 1.000 |
| Infusion-site reactions, n (%) | 15 (68.2) | 9 (64.3) | 6 (5.5) | 1.000 |
| Fever, n (%) | 4 (18.2) | 4 (28.6) | 0 (0.0) | 0.254 |
| Diffuse skin reactions, n (%) | 0 (0.0) | 0 (0.0) | 0 (0.0) | N/A |
| Dyspnoea, n (%) | 1 (4.5) | 1 (7.1) | 0 (0.0) | 1.000 |
| Sickness / dizziness / headache / nausea, n (%) | 3 (13.6) | 3 (21.4) | 0 (0.0) | 0.273 |
| Anaphylaxis, n (%) | 0 (0.0) | 0 (0.0) | 0 (0.0) | N/A |
| Fatigue, n (%) | 4 (18.2) | 4 (28.6) | 0 (0.0) | 0.254 |

Data were presented as number (percentage).

IVIg, intravenous immunoglobulin; SCIg, subcutaneous immunoglobulin; Ig, immunoglobulin.

# Supplementary Figures

Figure S1 Longitudinal normal immunoglobulin recipients over the past decade in Hong Kong


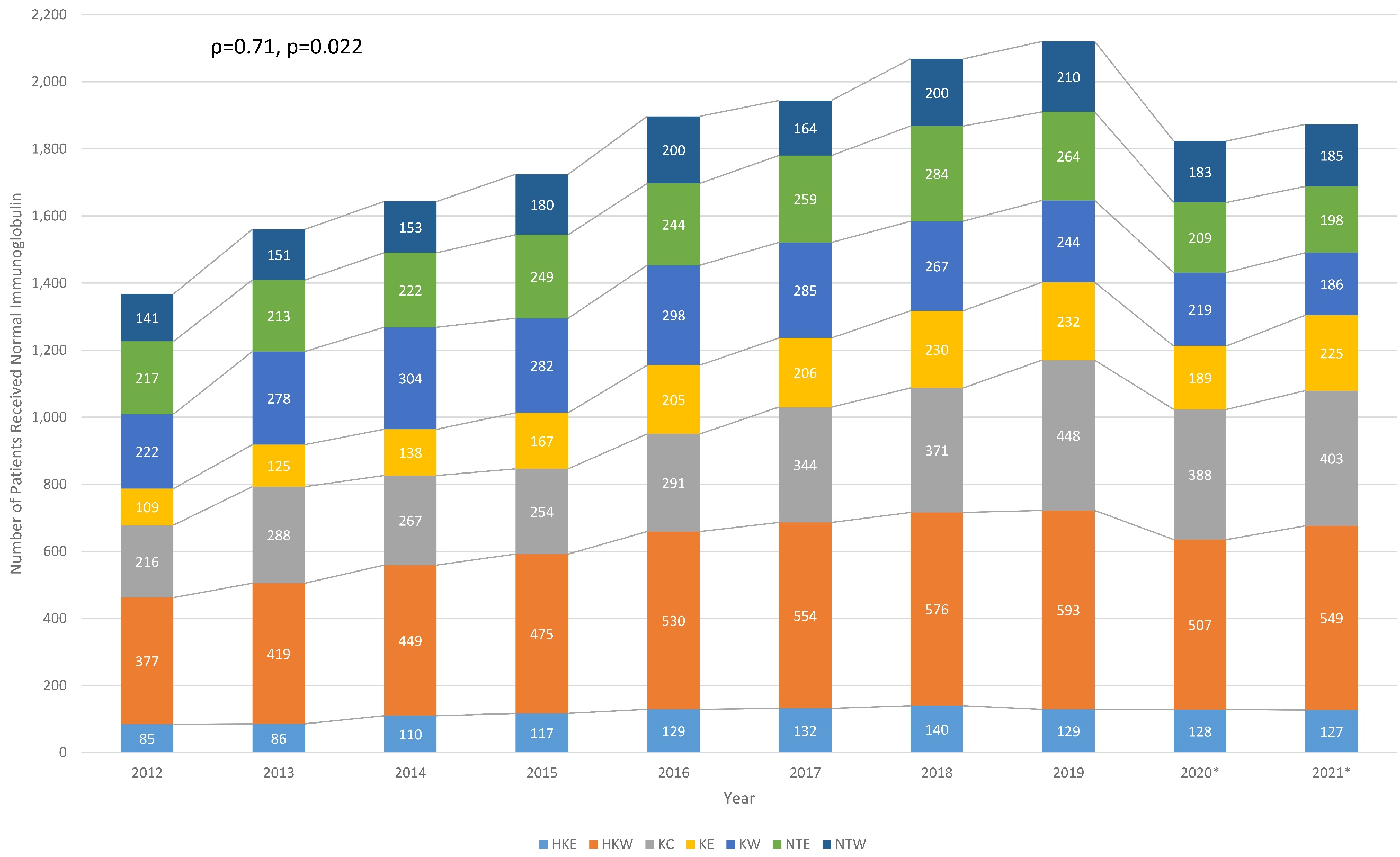


*Outbreak of COVID-19 in Hong Kong since January 2020, some of the healthcare services had been temporarily suspended upon waves of outbreak.

HKE, Hong Kong East Cluster; HKW, Hong Kong West Cluster; KC, Kowloon Central Cluster; KE, Kowloon East Cluster; KW, Kowloon West Cluster; NTE, New Territories East Cluster; NTW, New Territories West Cluster.

Figure S2 Longitudinal normal immunoglobulin expenditure over the past decade in Hong Kong


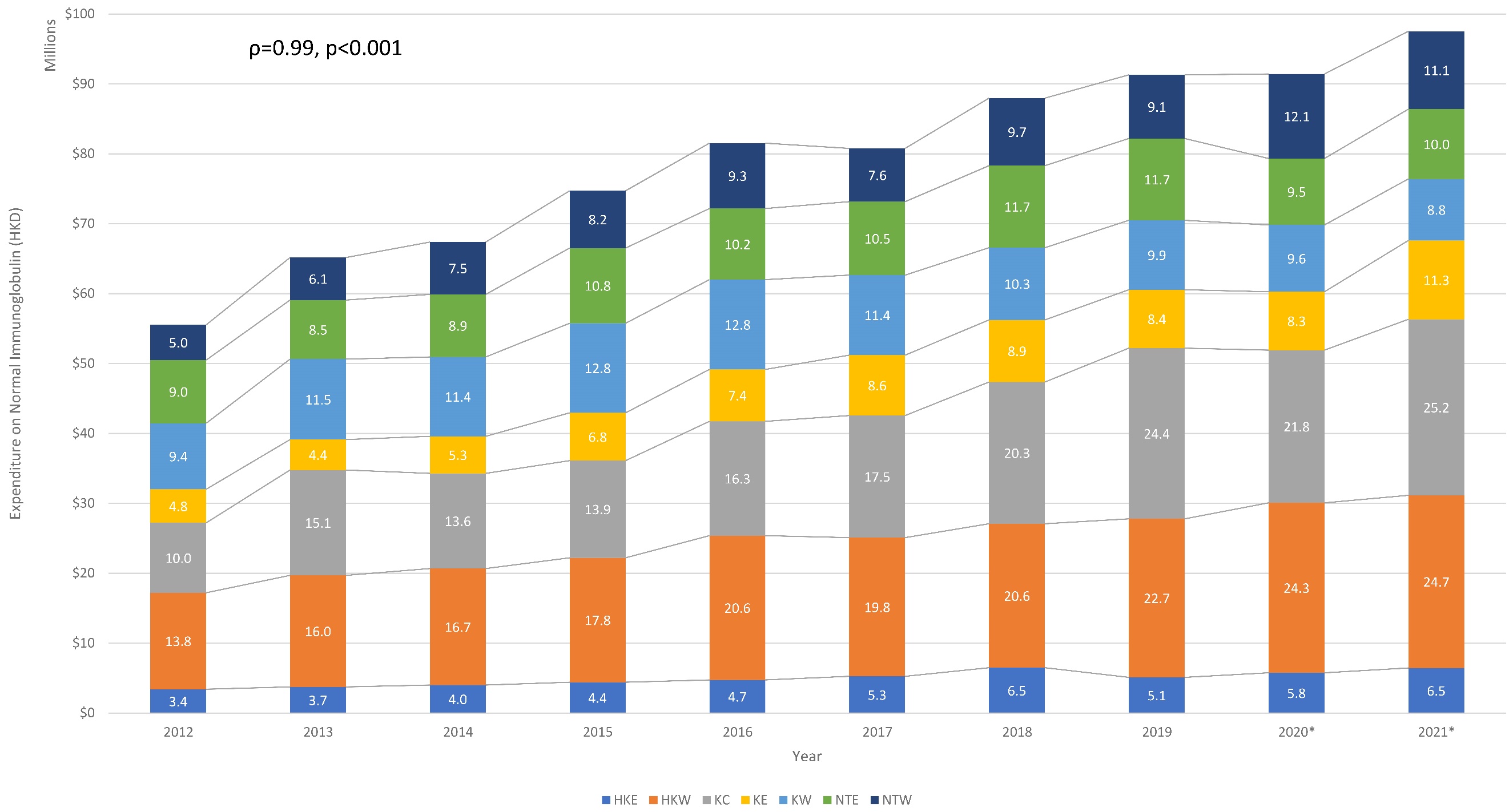


*Outbreak of COVID-19 in Hong Kong since January 2020, some of the healthcare services had been temporarily suspended upon waves of outbreak.

HKE, Hong Kong East Cluster; HKW, Hong Kong West Cluster; KC, Kowloon Central Cluster; KE, Kowloon East Cluster; KW, Kowloon West Cluster; NTE, New Territories East Cluster; NTW, New Territories West Cluster.

Figure S3 Breakdown of immunoglobulin treatment indications of adult patients at Queen Mary Hospital (Hong Kong) in 2021.


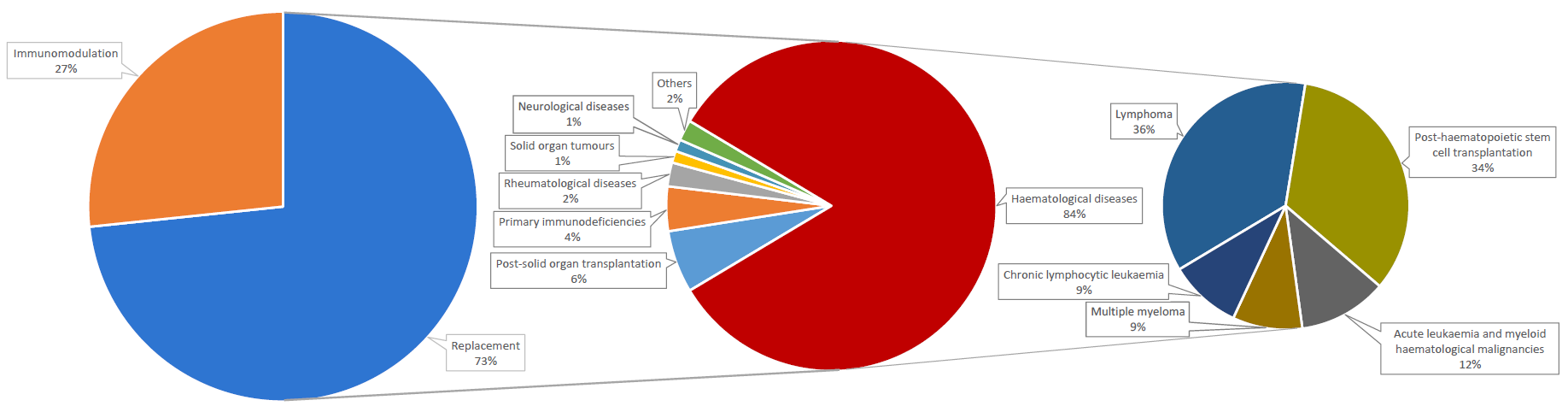

Supplement: Supplementary file 1 [file DataSheet_1.docx]
